# Lessons on the quality of tuberculosis diagnosis from standardized patients in China, India, Kenya, and South Africa

**DOI:** 10.1016/j.jctube.2019.100109

**Published:** 2019-06-07

**Authors:** Benjamin Daniels, Ada Kwan, Madhukar Pai, Jishnu Das

**Affiliations:** a Development Research Group, The World Bank, 1818 H Street NW, Washington, DC 20433, United States; b University of California at Berkeley, 2121 Berkeley Way, 5th Floor, Berkeley, CA 94720, United States; c McGill International TB Centre and Department of Epidemiology and Biostatistics, McGill University, 1020 Pine Avenue West, Montreal, QC H3A 1A2, Canada; d Centre for Policy Research, Dharma Marg, Chanakyapuri, New Delhi, India

**Keywords:** Quality of care, Tuberculosis, Standardized patients, Health care providers, Low- and middle-income countries

## Abstract

Standardized patients (SPs) are people who are recruited locally, trained to make identical scripted clinical presentations, deployed incognito to multiple different health care providers, and debriefed using a structured reporting instrument. The use of SPs has increased dramatically as a method for assessing quality of TB care since it was first validated and used for tuberculosis in 2015. This paper summarizes common findings using 3,086 SP-provider interactions involving tuberculosis across various sampling strata in published studies from India, China, South Africa and Kenya. It then discusses the lessons learned from implementing standardized patients in these diverse settings. First, quality is low: relatively few SPs presenting to a health care provider for the first time were given an appropriate diagnostic test, and most were given unnecessary or inappropriate medication. Second, care takes a wide variety of forms – SPs did not generally receive “wait and see” or “symptomatic” care from providers, but they received a medley of care patterns that included broad-spectrum antibiotics as well as contraindicated quinolone antibiotics and steroids. Third, there is a wide range of estimated quality in each observed sampling stratum: more-qualified providers and higher-level facilities performed better than others in all settings, but in every stratum there were both high- and low-quality providers. Evidence from SP studies paired with medical vignettes has shown that providers of all knowledge levels significantly underperform their demonstrated ability with real patients. Finally, providers showed little response to differences in patient identity, but showed strong responses to differences in case presentation that give some clues as to the reasons for these behaviors.

## 1 Introduction

Tuberculosis (TB) remains one of the most deadly diseases in the world. It accounted for an estimated 1.6 million deaths in 2017, overtaking the mortality attributed to HIV/AIDS [1]. That TB mortality is now higher than that due to HIV/AIDS reflects, in part, the effects of massive global investment in better diagnosis and antiretroviral therapy that has drastically reduced mortality due to HIV/AIDS. But it also reflects the fact that TB is a tenacious disease that is progressively becoming harder and more expensive to treat, as strains that display drug resistance to the standard treatment regimen become increasingly prevalent around the world.

Tackling TB requires renewed investments in every step of the care cascade that leads patients to successful diagnosis and treatment. In South Africa, the TB care cascade shows that just 53% of potential cases end up being successfully treated: 5% are lost at test access, 13% at diagnosis, 12% at treatment initiation and 17% at successful treatment completion [2]. The situation is even worse in India, with about 40% of patients in the public sector lost prior to diagnosis [3]. In China, TB prevalence has declined sharply from 215 in 1990 to 108 in 2010, thanks to improved treatment access for those who were already diagnosed [4], but the fraction of people with TB who remain undiagnosed is persistently high [5]. In countries like Kenya and South Africa, the majority of patients with TB are also infected with HIV (54% in Kenya and 70% in South Africa) [6], [7]. This further complicates diagnosis as high rates of HIV co-infection require that people with TB are tested for HIV and vice-versa.

Using data from these four countries, this paper summarizes recent research on the least well understood part of the care cascade-the quality of initial diagnosis and management of TB. This research is motivated by the fact that massive improvements in treating those who have already been diagnosed have not been matched with similar gains in the timely and accurate detection of individuals with TB in the first place [8], [9]. Difficulties in directly measuring the quality of primary care and providers’ diagnostic accuracy implies that we do not know how patients presenting with TB symptoms are managed at different stages of the disease. At this point, we cannot answer the simple question: “In Country X, what do the doctors do when a patient arrives, presenting with 2–3 weeks of cough, fever and night sweats?” Our relative ignorance about how TB patients are actually managed during this first critical interaction is particularly surprising, as delays at the diagnosis and treatment initiation stage allow patients to remain contagious and continue to spread the disease among their contacts. In fact, several systematic reviews show that TB is often diagnosed after about 2 months of delay, with several visits before diagnosis [10], [11].

In order to address this gap, researchers have developed and applied the method of standardized patients (SPs) to measure the quality of TB diagnosis and treatment in multiple settings. With the SP method, the research team recruits and trains a team of local people to present as patients with the same set of scripted symptoms (in this case, typical symptoms of TB) to a sampled set of health care providers [12]. Health care providers do not know when they are interacting with an SP, and therefore researchers are able to obtain accurate measures of the management that real patients presenting similar symptoms would receive.

By interacting in a real-life setting with health care providers, the SP method improves on “structural” quality measures, such as the availability of equipment and medicines, which have been shown to not predict actual clinical performance [13]. The SP method also has several advantages relative to quality measures based on clinical observation and chart abstraction. Because the underlying condition of the SP is fixed and known by design, the appropriateness of clinical action (or inaction) can be assessed against predetermined checklists; by contrast, any medical records that do exist may contain incomplete information on patients and recorded diagnoses may be incorrect. Standardizing the presentation further implies that an individual SP can present the “same” case to many providers, allowing valid comparisons across providers and locations. Since the SP presents unannounced to clinics, SP studies avoid “Hawthorne effects”, whereby providers change their behavior when they know they are being observed [12], [14]. Finally, training SPs to accurately recall each clinical encounter allows for the use of an extensive, detailed, structured exit questionnaire for each interaction to answer specific research questions.

Following previous large SP studies using asthma and angina cases in India [15], [16], our team extended the SP method to evaluate the quality of TB care in 2015 [12], [17]. This study showed that data generated from using the SP method for TB was (a) valid, with low detection rates and high agreement between audio recordings and structured questionnaires; (b) reliable, with key regularities documented in the data; and (c) informative, allowing researchers to understand both the overall quality of care and variation in care across different groups of providers. Since the pilot, research groups have applied the SP method to study the quality of TB care in large-N samples across multiple settings in India [18], [19], China [5], Kenya [12], and South Africa [20].

In this study, we summarize global findings using a combined sample of 3,086 SP-provider interactions using data from published studies in India [18], [19], China [5], South Africa [20] and Kenya [12]. We first focus on 1,861 provider-patient interactions with an SP portraying a classic case of a person who should be investigated for TB – the “Classic” scenario of presumed or suspected TB – which was used in all these studies. In the Classic scenario, the SP presented with 2–3 weeks of cough to a health care provider for the first time, and if prompted reveals additional symptoms such as fever and night sweats. We document the overall quality of care in these settings. We assess whether SPs received appropriate diagnostics in the form of TB testing and HIV testing. We also document whether they received unnecessary or harmful medicines, including fluoroquinolone antibiotics and steroids, which can mask TB symptoms and delay future diagnosis. The results demonstrate high variability in care quality across the settings and provider strata that SPs visited.

We then investigate the responsiveness of providers to variations in patient presentation, using additional data from the India study. We address whether provider behavior (a) varies according to the personal characteristics of the patient; and (b) is responsive to the information presented by the patient, versus being fixed by external constraints or incentives. The first examines the external validity of SP results to other patients and contexts. If the results vary enormously depending on the characteristics of the SP, conclusions are valid only for the specific group of SPs used in the study. The second question offers insights into the reasons for low average quality, a key direction that future research will need to tackle.

The India study provides two key avenues to approach these questions. We first use staff records from that study to assess the impact of the personal characteristics of the presenting individuals on management decisions by providers. Second, the study also included 1,225 additional interactions among the same set of providers, in which SPs reported to (a) have already undergone a chest X-ray and carried the (abnormal) image with them; (b) to have completed a laboratory sputum test and carried the (AFB-positive) result with them; or (c) to presented a classic history of multi-drug-resistant TB (treatment non-completion and symptom recurrence). We therefore use these variations to report how providers changed their management decisions when the patient presented different forms of information to them during the interaction.

## 2 Methods

In each of these primary studies, conducted after ethics approvals in each country, research teams recruited healthy individuals from the local population, who were then extensively trained to present TB cases to health care providers. Every SP was trained for several weeks to portray a single case presentation. The training focused on embedding the medical details of the case within the overall character of the SP in a consistent manner and further ensured that providers would not be able to distinguish the SPs from regular patients during the interactions.

Each study developed SP scripts detailing what information the SP was to give the provider at the beginning of the interaction, typically a short sentence in the local language such as: “*Doctor, I have a cough that is not getting better and some fever too.*” The scripts further specified exact responses to anticipated follow-up history questions from providers, including a scripted personal background, family and socioeconomic situation, and health history details such as smoking and alcohol use. SPs were coached on how to avoid unsafe situations and detection. The details of each study's SP recruitment, provider selection, and fieldwork implementation can be found in the respective primary studies and their online supplements [5], [18], [19]. Further general details on the SP method are available in a series of pilot and full-scale studies [15], [21], [22], [23], [24].

SPs were trained to recall all aspects of the interaction including the questions that the provider asked, the examinations they performed, the tests ordered and any treatment given. These were recorded in a structured questionnaire, developed through extensive consultations with a technical advisory group composed of local and global TB experts in each country, shortly after the SP left the provider's clinic. While SP presentations can vary contextually, all studies used the “Classic” presentation, characterized as “a case of presumed tuberculosis with 2–3 weeks of cough and fever”. In accordance with national and international guidelines as well as the recommendations of the studies’ advisory groups, the SP was typically judged to have been correctly treated if they received either a chest X-ray, a sputum AFB test or an Xpert MTB/RIF diagnostic. In the South African study, the study guidelines additionally required the provider to request an HIV test. No study penalized providers for provision of unnecessary or potentially harmful medications. However, since “correct” management is a complex contextual combination of offering appropriate care and avoiding inappropriate care, here we choose not to report an aggregated “correct” management for any setting, but instead report various outcomes separately for all studies. The public data that accompanies this paper allows researchers to further refine these measures in accordance with their specific requirements.

We report the management decisions observed in 1,861 Classic TB SP presentations completed between 2015 and 2018 in these four settings. For each study, we investigated: the proportion of “history checklist questions” completed (which varied by study); whether the provider ordered a chest X-ray, a sputum AFB or an Xpert MTB/RIF diagnostic; whether any medication was given or prescribed; use of any antibiotics; whether any anti-TB medication was used; the use of any contraindicated steroids or fluoroquinolone antibiotics; and whether an HIV test was ordered. This analysis reports unweighted proportions only, and the results reported here should not be considered to be nationally-representative comparisons for any of the constituent studies.

We also present separate estimates of quality of care outcomes for the Classic TB presentation by each study's primary sampling strata. In Kenya, SPs visited both public sector and private sector (for-profit and nonprofit) clinics in Nairobi; private sector providers account for a majority of all providers in the city. In South Africa, all interactions were conducted at public primary health care centres (PHCs). In India, all interactions were in the private sector and SPs visited “informal” and “formal” private sector clinics in urban Patna, and AYUSH and hospital-based private providers in Mumbai. Informal providers in Patna are those without MBBS (allopathic) degree qualifications; such providers account for 42% of all providers in Patna. AYUSH providers are those with a degree in alternative systems of medicine covering Ayurveda, Yoga, Unani, Siddhi and Homeopathy. In China, SPs visited village, township, and county-level public providers (the three levels of primary care in China) across Sichuan, Shaanxi, and Anhui provinces. Training and expertise in China increases at higher levels of care, moving from village to township to county. The exact details of the sampling strata and the sampling and visitation protocols are available in each study.

We also present additional results from the studies in urban India, where the team prepared and completed three additional case scenarios, resulting in an additional 1,225 SP interactions. SPs presented one of the following case scenarios:

• A case of presumed TB in a patient who has had 2–3 weeks of cough and fever. The patient has taken a broad-spectrum antibiotic (amoxicillin) given by another health-care provider for one week with no improvement. The SP also carries an abnormal chest X-ray suggestive of tuberculosis.
• Chronic cough with an AFB-positive sputum smear report for TB from a public health facility, highly suggestive of TB.
• Chronic cough, and, if asked, elaborates a history of previous, incomplete treatment for TB, which would raise the suspicion of multi-drug-resistant TB.

These additional case presentations were piloted in a previous study to ensure that the presentation with medical records was not considered unusual in the context [12]. These presentations were each conducted among a statistically comparable subset of the providers who also saw an SP giving the Classic TB presentation. This sampling design allows differences between the actions of the providers to be attributed to the change in case presentation rather than other confounding factors. Therefore, we compare these additional cases separately to the Classic TB case presentation on the same outcome measures.

## 3 Results

### 3.1 Management of the classic TB case presentation

**Fig. 1:**
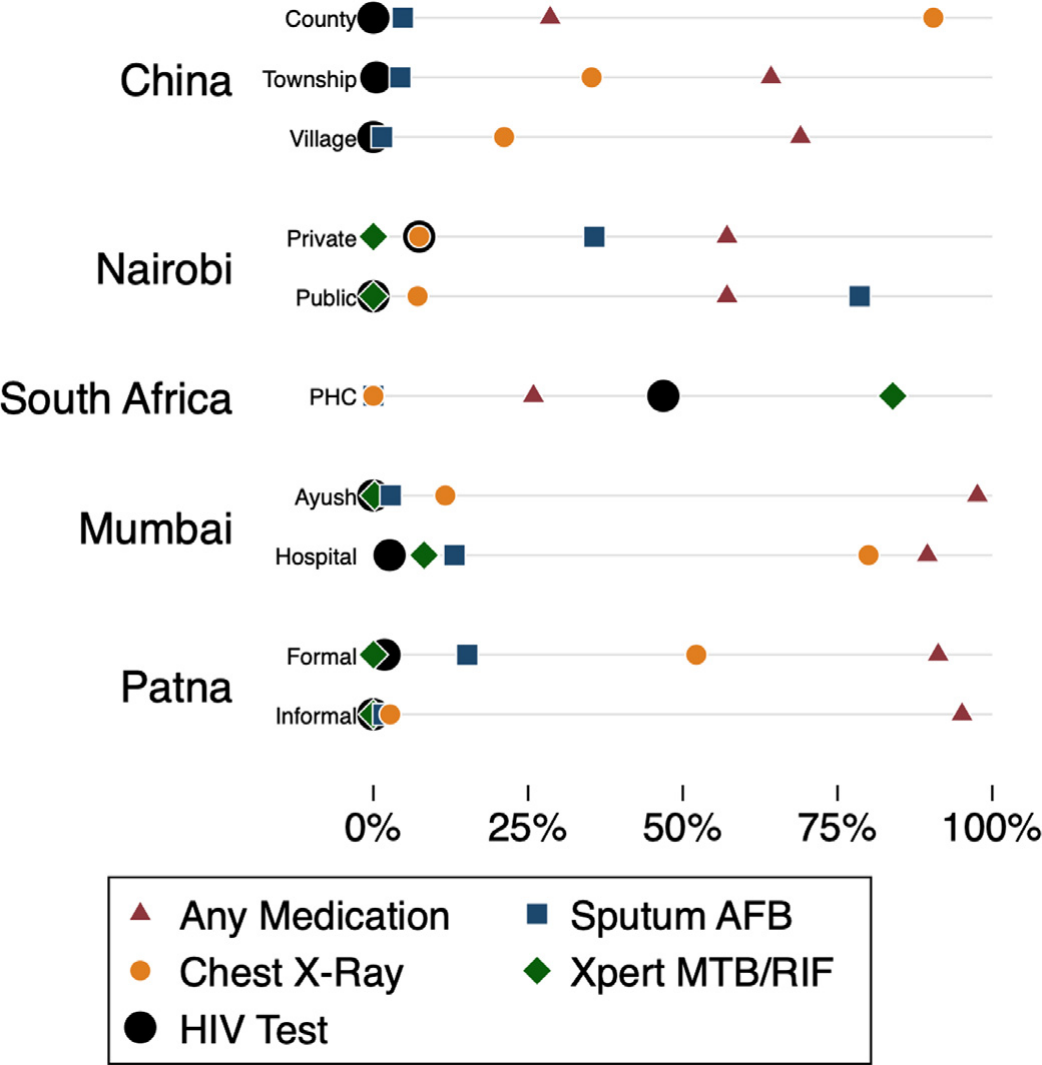
Classic TB case management by study and strata *Note:* This figure reports the overall proportion of SPs presenting the Classic TB case in each study who received each of the indicated management decisions by the provider. Number of observations: China County (21), Township (207), Village (71); Nairobi Private (28), Public (14); South Africa Public PHC (143); Mumbai Private Ayush (499), Private Hospital (305); Patna Private Formal (389), Private Informal (184). AFB: Acid-Fast Bacillus; MTB/RIF: Mycobacterium Tuberculosis/Rifampicin.

We report three main findings for the Classic case presentation. First, the use of appropriate TB testing varies widely across the study population, ranging from 4% among informal private clinics in Patna to 90% in county-level public hospitals in China. As can be seen in Fig. 1, testing frequency was substantially higher in higher-level clinics than in lower-level clinics (in China), in formal clinics and hospitals than in informal clinics and AYUSH providers (in India), and public than in private clinics (in Kenya). Chest X-rays, recommended in 36% of interactions, were the most popular TB test ordered in India and China; in Nairobi both public and private sector providers preferred sputum smears, which were ordered in 50% of interactions. At the time of these studies, Xpert MTB/RIF testing was just becoming available in most settings and was observed only among South Africa PHCs (84%) and Mumbai providers in hospitals (3%). In South Africa, test orders were not directly observed, but Xpert testing is in place nationally, so all sputum collection was recorded as Xpert. [25] HIV testing was rare in most settings – less than 5% in all study strata except South Africa PHCs, in which 47% of SPs were ordered HIV tests.

All types of providers (although not all providers) were observed to prescribe some kind of medication to the SP (83% of interactions), and these were likely to be demonstrably unnecessary or harmful. Table 1 highlights three classes of medications that are of special interest for TB treatment: broad-spectrum antibiotics, fluoroquinolone antibiotics and steroids. These medications either have adverse public health implications or are contraindicated in possible TB cases because they may mask symptoms and delay further diagnosis. We also documented the use of unlabelled, traditional, and homeopathic medicines whose provenance is unknown; the use of such medicines are problematic as they convey no information to the patient. Strikingly, cases where none of these medicines were used are in the minority everywhere. We observe every possible combination of these medications in the data at least once, with providers in every study strata except South Africa PHCs prescribing or administering antibiotics in more than half of cases.

**Table 1.** Use of unnecessary and contraindicated medications for the Classic TB case.

	China County	China Township	China Village	Nairobi Private	Nairobi Public	South Africa PHC	Mumbai Ayush	Mumbai Hospital	Patna Formal	Patna Informal
None	15 (71%)	75 (36%)	22 (31%)			132 (92%)	13 (3%)	49 (16%)	43 (11%)	10 (5%)
Antibiotics	1 (5%)	15 (7%)	5 (7%)			11 (8%)	2 (<1%)	76 (25%)	80 (21%)	14 (8%)
Quinolones							1 (<1%)	4 (1%)	13 (3%)	3 (2%)
Steroids								5 (2%)	3 (1%)	
Unlabelled	3 (14%)	13 (6%)	18 (25%)	13 (46%)	6 (43%)		226 (45%)	33 (11%)	13 (3%)	73 (40%)
Antibiotics + Quinolones								1 (<1%)	8 (2%)	1 (1%)
Antibiotics + Steroids		1 (<1%)						28 (9%)	63 (16%)	11 (6%)
Antibiotics + Unlabelled	2 (10%)	85 (41%)	25 (35%)	13 (46%)	8 (57%)		143 (29%)	67 (22%)	62 (16%)	23 (13%)
Quinolones + Steroids		1 (<1%)						4 (1%)	18 (5%)	3 (2%)
Quinolones + Unlabelled		3 (1%)	1 (1%)	1 (4%)			15 (3%)	6 (2%)	18 (5%)	7 (4%)
Steroids + Unlabelled		1 (<1%)					34 (7%)	11 (4%)	8 (2%)	9 (5%)
Antibiotics + Quinolones + Steroids								2 (1%)	10 (3%)	
Antibiotics + Quinolones + Unlabelled		11 (5%)					4 (1%)	2 (1%)	12 (3%)	3 (2%)
Antibiotics + Steroids + Unlabelled		1 (<1%)		1 (4%)			54 (11%)	13 (4%)	27 (7%)	17 (9%)
Quinolones + Steroids + Unlabelled							7 (1%)	3 (1%)	6 (2%)	7 (4%)
Antibiotics + Quinolones + Steroids + Unlabelled		1 (<1%)						1 (<1%)	5 (1%)	3 (2%)
Number of Observations	21	207	71	28	14	143	499	305	389	184

Note: Medications from each interaction were ex-post coded by name to correspond to ATC code classifications, the WHO system for categorizing medications according to their purpose and action. This figure reports the whole number of SP interactions in which each combination of the following medicine classes were observed to have been given to the patient, and the corresponding proportion of interactions in that sampling stratum. The categories are: broad-spectrum antibiotics (other than fluoroquinolones), defined as ATC codes beginning with J01 but not J01M; fluoroquinolone antibiotics, defined as ATC codes beginning with J01M; steroids, defined as ATC codes beginning with H02, R01, or R03; and unlabelled medications, for which the field team was unable to identify generic ingredients (including homeopathic, traditional, and herbal medications, but excluding vitamin and mineral preparations). Percentages may not add to 100% due to rounding.

One potential explanation for the low adherence to national and international standards of TB care is that the healthcare providers in these samples did not have the requisite training or knowledge to follow the protocols correctly. Therefore, in studies in Delhi and China, researchers have sent trained enumerators to the same providers some weeks after the completion of Classic TB SP visits and asked the providers to evaluate a hypothetical patient who presents with the exact same symptoms and scripts as the SPs did [5], [12]. The use of such medical vignettes had been validated in India previously and shown to produce reliable measures of medical competence or knowledge [26]. In both these paired vignette studies, the same providers were far more likely to recommend management options consistent with standards of TB care in the vignettes as they were with identical SPs. In China, the use of chest X-rays or sputum tests was 47 percentage points higher in vignettes than in SP interactions, and in Delhi it was 62 percentage points higher. Similarly, in both studies, providers gave more unnecessary medications to the SPs than they said they would during the medical vignettes. This phenomenon, labelled the “know-do” gap, has been replicated in multiple studies and cites since it was first highlighted in studies from India and Tanzania [27], [28].

**Fig. 2:**
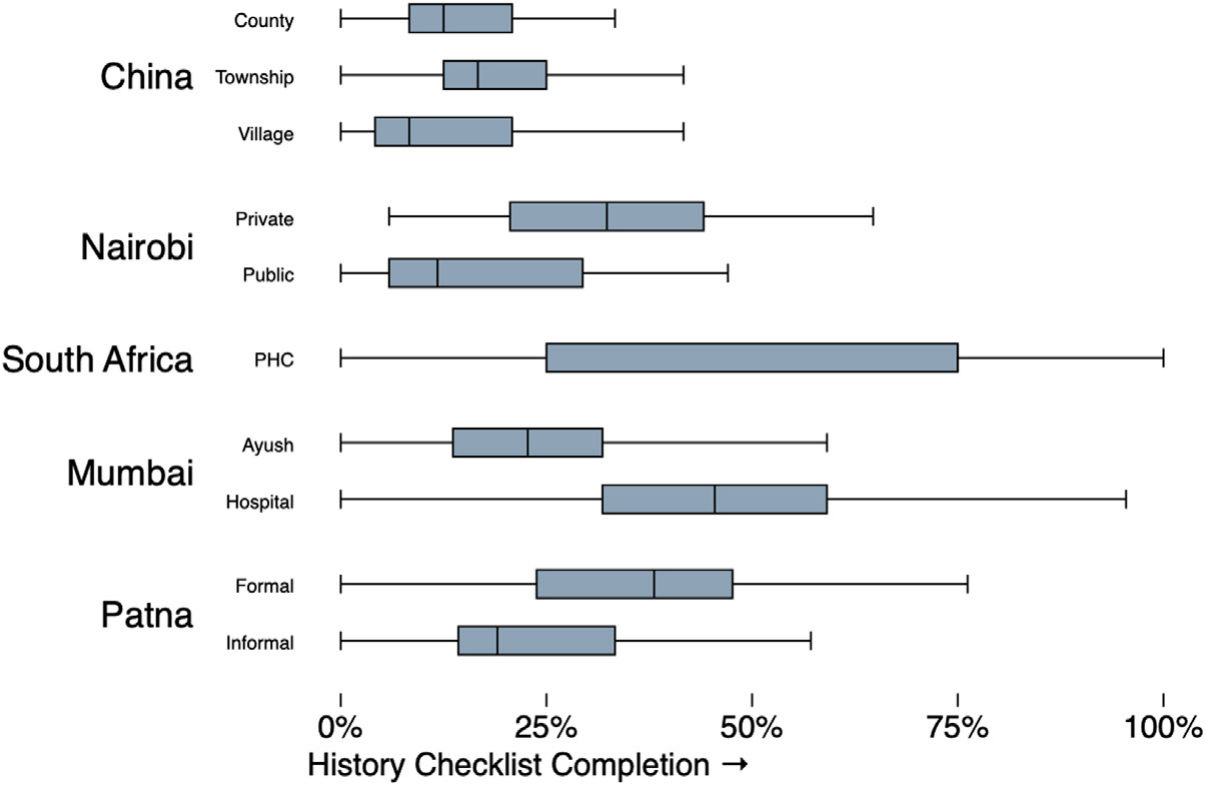
History checklist completion for the Classic TB case: variation by study and strata *Note:* Using the context-specific measure of history checklist items, this figure illustrates the range of item completion within each study and sampling strata. Number of observations: China County (21), Township (207), Village (71); Nairobi Private (28), Public (14); South Africa PHC (143); Mumbai Ayush (499), Hospital (305); Patna Formal (389), Informal (184). History checklist questions in each study are a subset of: Duration of Cough, Sputum, Past TB, Family TB, Blood in Sputum, Cough Throughout Day, Fever, Fever Type, Family or Family with Similar Symptoms, Chest Pain, Loss of Appetite, Lost Weight, Wheezing, Difficulty Breathing, Smoking, Alcohol History, Taken Medicines for Illness, Diabetes, HIV/AIDS, Age, TB Suspicion, MDR-TB Suspicion, High blood pressure or hypertension, Weakness, Night Sweats.

We used adherence to an essential checklist of history questions to examine variation in quality of care within every setting and strata, using the checklist questions reported for the underlying study (these are not comparable across settings). Fig. 2 illustrates this variation. While the practices of different groups in each study differ *on average*, there is a broad range of observed provider behavior within each group as well. In no study did any provider type strictly dominate all the others. Instead, these provider type and qualification classifications were weakly associated with provider behavior, and the variations are wide within each strata. Every strata group contains providers who complete many checklist items as well as providers who complete very few.

### 3.2 Provider response to variation in patient presentation

These results raise key questions about how providers respond to patient presentation. The first question relates to the external validity of SP studies. Since many SP studies use a small number of individual SPs drawn from specific backgrounds in order to be suitable for data collection, the findings from these studies may not be applicable to general patient populations. For instance, SPs typically have at least secondary education, whereas many TB patients may have education at the primary or less than primary level. If providers behavior varies by the personal characteristics of SPs, each of these studies is ultimately limited to the specific group of SPs that was deployed. The India study used a relatively larger number of individual SPs in order to accommodate the scale of the study, and we use this design to investigate the role that individual SP identities played in their treatment outcomes.

**Fig. 3:**
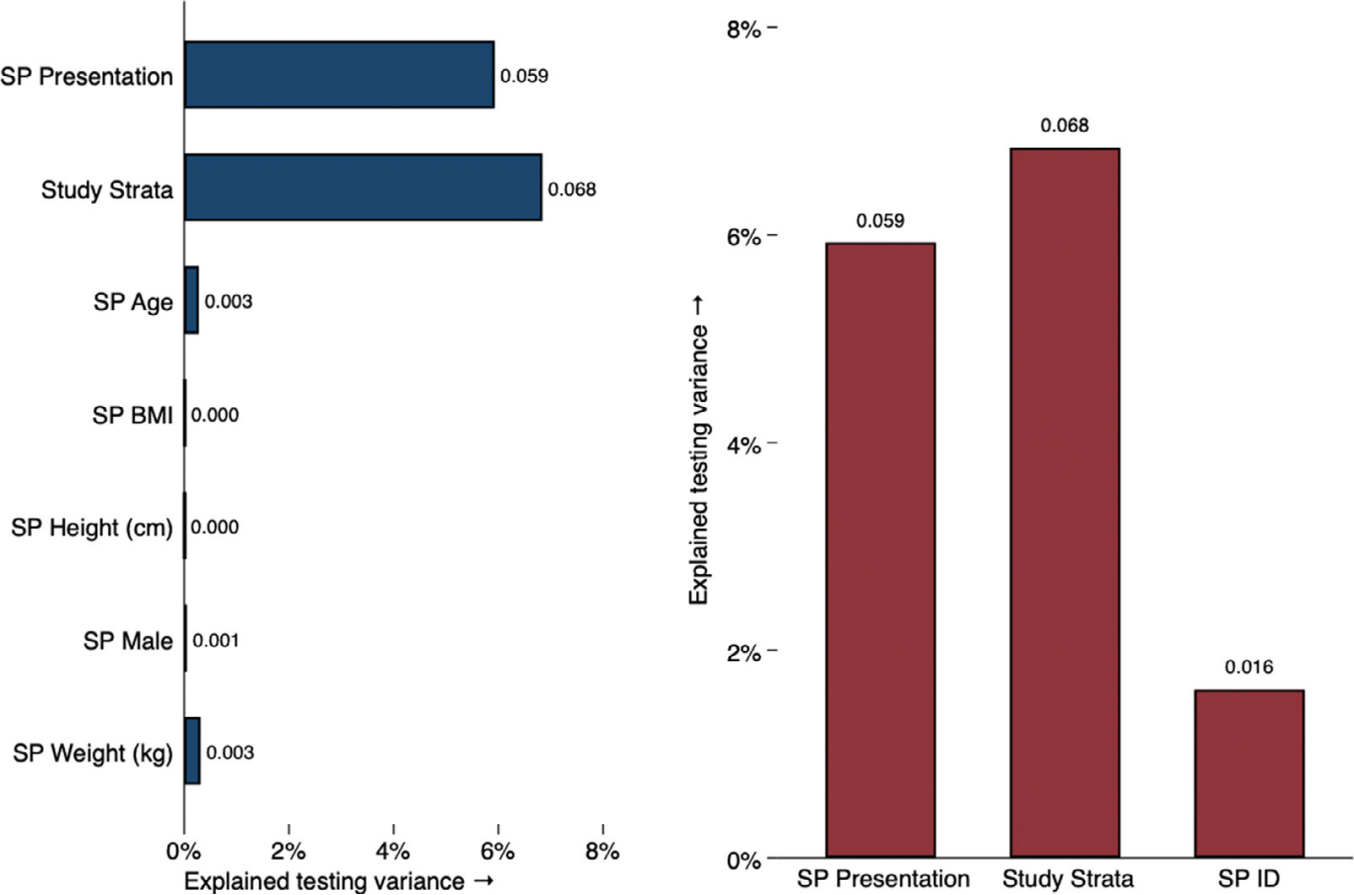
ANOVA analysis of variation in TB testing explained by patient presentation *Note:* This figure reports the sequential-ANOVA contribution of SP characteristics to the explained variance of receiving at least one of the following TB tests: a chest X-ray, a sputum AFB smear, or an Xpert MTB/RIF test. The binary outcome is first regressed on SP presentation and study location and strata, and in the first panel, individual characteristics are sequentially added and the improvement in explained sum of squares reported. In the second panel, the entire set of individual SP identity indicators is added to the regression and its overall contribution to explained variation in TB-testing outcomes is reported. AFB: Acid-Fast Bacillus; MTB/RIF: Mycobacterium Tuberculosis/Rifampicin.

Fig. 3 uses the individual characteristics of the included SPs we hired in an ANOVA analysis. We added these characteristics sequentially to a regression model after controlling for the case presentation, study setting and provider type. The first panel shows that SP age, gender, height, weight, and BMI do not significantly contribute to explaining variation in TB testing. This is of independent interest, as there is a concern that doctors change their behavior if SPs do not conform with their view of what a TB patient looks like; in our data we do not find strong evidence for this view. In the second panel, we use an individual indicator variable (dummy) for every SP used in the study to fully test for differences across all specific individuals. Although we cannot rule out the hypothesis that SP identity indicators are jointly significant – they seem to have some effect – we observe that the overall variation in outcomes due to all SP characteristics is (at most) 13% of the amount of variation explained by case presentation, city, and strata indicators.

The fact that health care providers do not alter their behavior substantially depending on the background of the SP does not necessarily imply that their behavior is invariant to the specific case presented by the SP. In the research on private providers, one question of particular interest in is the role of financial incentives. A potential explanation for poor quality is that providers do not want to disclose that the patient has TB due to the fear that the patient will then seek care elsewhere, hurting the revenue of the provider. Indeed, SP studies combined with provider knowledge surveys have repeatedly demonstrated that health care providers typically do far less for patients than they have the knowledge to do.

**Fig. 4:**
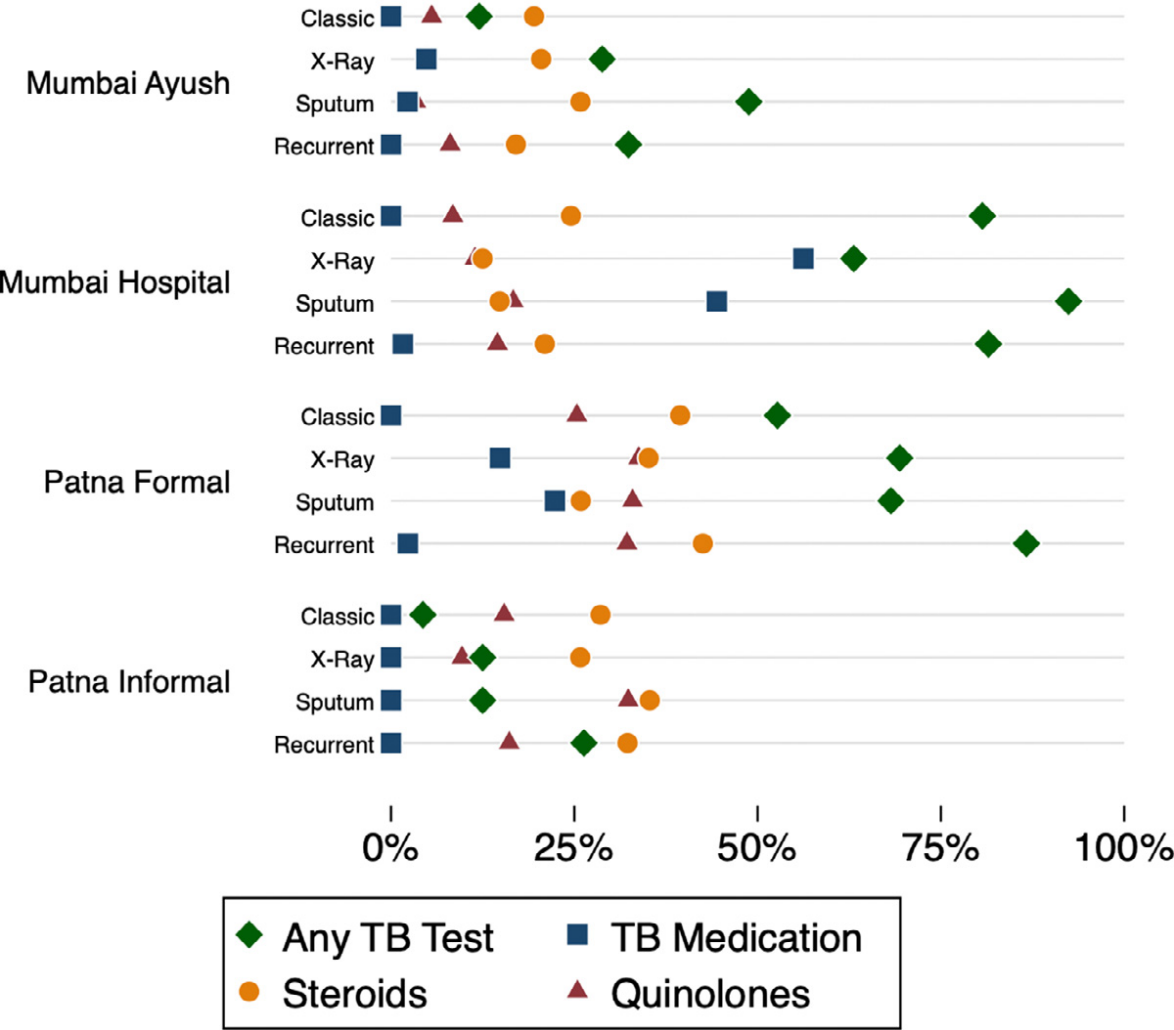
TB-related testing, anti-TB medication, and contraindicated medication use by case presentation at private providers in urban India for all case presentations *Note:* This figure reports the usage of laboratory testing, anti-TB medication, fluoroquinolone antibiotics, and steroids across each study strata for four case presentations. These were the Classic TB presentation; the X-ray presentation; the sputum report presentation; and the MDR/recurrence presentation. Number of observations: Mumbai Ayush Classic (499), X-ray (125), Sputum (125), MDR (247); Mumbai Hospital Classic (305), X-ray (122), Sputum (79), MDR (81); Patna Formal Classic (389), X-ray (98), Sputum (110), MDR (120); Patna Informal Classic (184), X-ray (40), Sputum (40), MDR (38). Medications from each interaction were ex-post coded by name to correspond to ATC code classifications. This figure reports the proportion of SP interactions in which each of the following medicine classes were observed to have been given to the patient: fluoroquinolone antibiotics, defined as ATC codes beginning with J01M; and steroids, defined as ATC codes beginning with H02, R01, or R03.

The India study addressed this explicitly by varying the information that the SP made available to the provider in a given interaction. The idea was that if the SPs presented diagnostic information that they were not able to interpret themselves, providers driven only by financial incentives would continue to behave in the same way as in the presentations with less available information. Alternatively, if the providers had in fact had difficulty diagnosing the SPs, their behavior should change in response to the new information. SPs presented three additional TB case scenarios to a randomized subset of the same providers, allowing direct comparability between management in those cases and in the Classic case. In each of these cases, the SP presented additional information in the form of a specific TB test such as an abnormal chest X-ray or an AFB-positive sputum smear report. The first panel in Fig. 4 summarizes TB-related management across all case presentations for each of the sample strata, highlighting the use of TB testing (chest X-ray, sputum AFB smear, or Xpert MTB/RIF); anti-TB medication; and contraindicated steroids and fluoroquinolone antibiotics.

For all types of providers, the use of TB testing was higher when the SP carried an AFB-positive sputum smear report, even though the SPs clearly indicate that they do not know what results the test implies. This is also true for the case presenting the abnormal chest X-ray in all but one strata. We also report overall rates of fluoroquinolone and steroid usage for each of the case presentations. As in the overall data for the Classic TB case, there are no clear patterns and wide variation in usage across both strata and case presentation.

## 4 Discussion

### 4.1 Strengths and limitations of SPs for tuberculosis

Following our first study validating the use of SPs to measure the quality of TB care, researchers have successfully deployed SPs in large provider samples across multiple countries. These studies are tailored to the country context, and therefore differ in their sampling schemes and providers covered; consequently, we have been able to show that the SP methodology can be successfully used for a variety of providers (public, private, formal, informal, etc.) in various settings.

The methodology has proved to have a variety of strengths and weaknesses in policy-relevant applications. In terms of strengths, it remains the only method by which complete information about provider behavior can be acquired from a targeted sample for a specific case scenario. This is essential to construct measures of access to quality care at a population level; estimating the frequency of antibiotic use and misuse; supporting and improving the function of disease control programs; monitoring patient safety measures; and evaluating training programs.

With greater use, the limitations to this method have also become clearer. SPs cannot measure the quality of care received over the entire treatment phase of TB care, for example, because they are not in fact ill. SPs have not yet been used to construct standardized measures that include follow-up visits to providers. SPs also remain limited by nature in the patient presentations that are feasible in terms of identities and conditions; and utilizing SPs in more advanced medical and insurance systems poses major operational challenges to SP studies since they lack “real” identities for these systems. Currently, we also cannot construct full care cascades for SPs following a misdiagnosis in the first interaction. Suppose providers ask SPs to return if they are not improving – what happens next? In the studies summarized here, the SPs simply never went back. Our team has now carried out a small (unpublished) field pilot to investigate the ability of the SP method to be useful in such cases; the principal difficulty is that a single initial SP presentation can quickly morph into various “un-standardized” cases as each SP will likely have received a different treatment in the first interaction.

### 4.2 Global findings from the use of SPs for tuberculosis

Several important patterns in quality of care have become apparent from SP studies focusing on TB. First, appropriate TB testing increases as expected at higher levels of care-formal providers and hospitals in India and county doctors in China were more likely to appropriately test patients for TB when symptoms warranted. Second, even as appropriate testing increases at higher levels of care, the inappropriate use of medicines does not decline. In fact, formal providers and hospitals in India, and township clinics in China were just as likely to use antibiotics and quinolones as their less-qualified counterparts. Similarly, although care was better in the public sector in Nairobi than the private sector, medication use was identical. Third, even though higher levels of care are associated with greater use of appropriate testing, there is always wide variability in quality within every setting and strata. There were always *some* informal providers who did more than *some* formal providers in India; *some* village clinics that did more than *some* township hospitals in China; and *some* private providers that did more than public providers in Nairobi, for example.

One important question these studies raise is whether it is fair to expect health care providers to recommend TB tests on a new patient's very first visit. Cities like Patna and Mumbai in India are polluted and conditions associated with chronic coughs are therefore common. In these situations, it may be appropriate for providers to recommend cough suppressants and move to TB testing if the symptoms do not subside. We are sympathetic to this critique. However, two patterns in the data lead us away from this specific interpretation. First, providers do not follow a general “alternate” protocol where medicines are used for symptomatic relief prior to further testing. Instead, we find widespread use of all types of medicine combinations, including those that can lead to antibiotic resistance and can mask TB symptoms in the future. Second, providers at higher levels of care are always more likely to recommend appropriate treatments. This suggests that even if our benchmarks of appropriate treatment are unrealistic, they remain helpful in ranking different types of providers in a manner that is consistent with their practice setting and qualifications. Rather than limiting analysis to a binary definition of right and wrong, detailed SP data can be used to classify providers across a multidimensional quality spectrum, and these classifications can be further refined by using auxiliary information such as the completion of checklist items.

A second question raised by these studies is the underlying reasons for frequent mismanagement. One possibility is that providers do not have the knowledge to diagnose patients appropriately. However, studies from India and China were able to directly compare performance with SPs to tests of knowledge on the same conditions and demonstrate a “know-do gap” in the data. Providers misdiagnosed and mismanaged SPs at a much higher rate than what tests of their knowledge would suggest. Additionally, in India, giving additional diagnostic information to the same providers in the alternate presentations led to higher rates of TB-related management, suggesting that there is at least some gap preventing providers from recognizing the condition in real practice. Further study in Kenya and South Africa is needed to determine whether such patterns hold in those settings as well.

Another possibility is that providers-particularly those in the private sector-deliberately misdiagnose patients to maximize their revenue when patients return. Our data from case variants where the SP provides a positive test result show that even if revenue considerations are part of the explanation they cannot be the *only* reason for frequent misdiagnosis. When SPs carry a positive test result, misdiagnosis declines even if providers take a financial loss from doing so. Since SPs indicate to the provider that they cannot interpret the test result, the providers should not have changed their behavior if their only aim was to maximize their revenues. These results suggest instead that providers face systematic (yet currently unidentified) barriers in their attempts to diagnose TB patients that are unrelated to knowledge or financial incentives. What these barriers are requires further research.

A final concern with SP studies is that the individual identity of the SPs could dramatically affect study results (and, therefore, outcomes for real patients), and many studies to date have used only a small number of individual people as SPs. In these studies, SPs were hired from a healthy working population and selected for their ability to accurately and reliably perform a very difficult job in the field. It could be that providers are being systematically misled by the appearance or behavior of these SPs, leading them away from an accurate diagnosis. However, as the same SPs were used for all providers, the higher frequency of appropriate treatment among formal providers and at higher levels of care mitigates against this possibility: “better” providers who asked more TB-relevant questions were more likely to behave as if the SPs actually had TB. In addition, by using detailed staff information from the large study in India, we are able to look directly at the correlations between quality of care and anthropometric data on SP gender, height, weight, BMI and age and by comparing individual SPs directly. The personal characteristics of SPs have little systematic effect on the quality of the care provided. Though we cannot rule out individualized effects completely, we show that they are small relative to differences across providers and cases and seem to be nothing more than the normal variation that any diverse patient population would entail – there is, after all, no perfect “Classic Case”.

With these broad-based results, we believe that SP studies therefore satisfy multiple demanding requirements for the reliable measurement of diagnostic and treatment accuracy for TB. We have made substantial progress in addressing several open questions that have been raised following our initial validation study. Additionally, our fieldwork has led us to believe that while SP studies have high start-up costs in terms of initial staffing and provider mapping, when studies are geographically concentrated or maintained over time, the marginal costs of additional investigations using SPs are significantly lower than in smaller studies.

### 4.3 Policy relevance of SP study findings for tuberculosis

To date, the problem of tuberculosis management has been framed and researched as a post-diagnosis question. It is only now with the development of TB care cascades, studies of the delays in diagnosis, and the use of the SP methodology that we are starting to discover that the diagnosis stage itself can pose a major hurdle towards effective TB care. Since the problem has surfaced so recently, what to do about it is less clear, particularly as multiple studies have shown that changing provider behavior at this stage is quite difficult.

The main benefit of the SP method thus far has been to bring the problem of diagnosis front and center in global health discussions. We are very much in the beginning stages of understanding how the method can then be embedded in quality improvement efforts around the world, but there are emerging positive signs. As one example, our research team has worked closely with the Gates Foundation to assess a program that networked private providers and worked intensely with them over a period of several years to improve quality of care and increase TB notifications. Early results from this program suggest that significant improvements in TB diagnosis were achieved in both the cities where this program was trialed [29]. As a second example, one of us (Pai) has been involved with the World Health Organization to bring the problem of diagnosis to the forefront and this collaboration has led to the development of the first essential diagnostics list [30].

Achieving further progress in the future now requires us to urgently understand why misdiagnoses are so frequent at first contact. It also requires us to understand and why so many providers continue to use a cocktail of antibiotics, steroids, and quinolones to treat patients with 2–3 weeks of cough, when these approaches may mask TB symptoms. We must understand why providers do not typically immediately suggest a TB-sensitive test like an X-ray in these cases, if only to rule out TB. We suspect that the widespread use of incorrect medicines may be linked to financial incentives, and this is a further area to explore, especially given the rapid rise of anti-microbial resistance in countries like India. Indeed, ethnographic and qualitative research now suggests that multiple pressures drive the widespread empirical practices observed, including the use of medications as diagnostic tools, a desire to provide rapid symptom relief to patients, a desire to manage illness cost-effectively, uncertainty about the presentation of TB, and uncertainty about the accuracy of available TB tests [31].

## Ethical approvals

Each study presented in this paper was granted ethical clearance from institutional review boards (China: Institutional Review Boards at Stanford University, United States (Protocol Number 25904) and Sichuan University, China (Protocol Number K2015025); India: McGill University Health Centre, Canada (REB No. 14-137-BMB) and the Subcommittee for the Ethical Approval of Projects at the Institute for Socioeconomic Research on Development and Democracy in Delhi, India; Kenya: the review board at African Medical and Research Foundation (AMREF), Reference AMREF-ESRC P94/2013, with additional clearances from the Ministry of Health, Government of Kenya and each county in which the facilities were located; South Africa: Research Ethics Committee for Human Research (Humanities) at Stellenbosch University, South Africa (HS1096/2014-REC)). Since we rely on data produced from these different studies, a separate ethical approval was not obtained for the analysis in this paper.

## Declarations of interest

None

## Competing interests

None of the authors have any competing interests to disclose.

## Disclaimer

The findings, interpretations, and conclusions expressed here are those of the authors and do not necessarily represent the views of the World Bank, its executive directors, or the governments they represent.

## Data availability statement

Individual de-identified interaction data, including data dictionaries, will be available. All variables needed to re-create the results reported in this article will be included, as will the code required to reproduce these results. Data will be available indefinitely upon publication to anyone who wishes to access the data for any purpose. The data and code can be accessed at https://github.com/qutubproject/jclintb2019.

## Appendix Supplementary materials

Image, application 1

## References

[1] World Health Organization, Others. Global tuberculosis report2018. 2018.

[2] Naidoo P., Theron G., Rangaka M.X. (2017). The South African tuberculosis care cascade: estimated losses and methodological challenges. *J Infect Dis*.

[3] Subbaraman R., Nathavitharana R.R., Satyanarayana S. (2016). The tuberculosis cascade of care in India's public sector: a systematic review and meta-analysis. *PLoS Med*.

[4] World Health Organization. Tuberculosis in China. accessed 8 May2019. http://www.wpro.who.int/china/mediacentre/factsheets/tuberculosis/en/.

[5] Sylvia S., Xue H., Zhou C. (2017). Tuberculosis detection and the challenges of integrated care in rural China: a cross-sectional standardized patient study. *PLoS Med*.

[6] Odhiambo J., Kizito W., Njoroge A. (2008). Provider-initiated HIV testing and counselling for Tb patients and suspects in Nairobi, Kenya. *Int J Tubercul Lung Dis*.

[7] Karim S.S.A., Churchyard G.J., Karim Q.A., Lawn S.D. (2009). HIV infection and tuberculosis in South Africa: an urgent need to escalate the public health response. *Lancet*.

[8] Schnippel K., Ndjeka N., Maartens G. (2018). Effect of bedaquiline on mortality in South African patients with drug-resistant tuberculosis: a retrospective cohort study. *Lancet Resp Med*.

[9] Ahmad N., Ahuja S.D., The Collaborative Group for the Meta-Analysis of Individual Patient Data in MDR-TB treatment–2017 (2018). Treatment correlates of successful outcomes in pulmonary multidrug-resistant tuberculosis: an individual patient data meta-analysis. *Lancet*.

[10] Sreeramareddy C.T., Qin Z.Z., Satyanarayana S., Subbaraman R., Pai M. (2014). Delays in diagnosis and treatment of pulmonary tuberculosis in India: a systematic review. *Int J Tubercul Lung Dis*.

[11] Sreeramareddy C.T., Panduru K.V., Menten J., Van den Ende J. (2009). Time delays in diagnosis of pulmonary tuberculosis: a systematic review of literature. *BMC Infect Dis*.

[12] Das J., Kwan A., Daniels B. (2015). Use of standardised patients to assess quality of tuberculosis care: a pilot, cross-sectional study. *Lancet Infect Dis*.

[13] Das J., Gertler P.J. (2007). Variations in practice quality in five low-income countries: a conceptual overview. Health Affairs.

[14] Leonard K., Masatu M.C. (2006). Outpatient process quality evaluation and the Hawthorne Effect. *Soc Sci Med*.

[15] Das J., Holla A., Das V., Mohanan M., Tabak D., Chan B. (2012). In urban and rural India, a standardized patient study showed low levels of provider training and huge quality gaps. Health Affairs.

[16] Das J., Holla A., Mohpal A., Muralidharan K. (2016). Quality and accountability in health care delivery: audit-study evidence from primary care in India. *Am Econ Rev*.

[17] King, J. et al. How to do (or not to do)... using the standardised patient method to measure clinical quality of care in LMIC health facilities. Health Policy and Planning Unpublished. 10.1093/heapol/czz078 PMC6904318 31424494

[18] Daniels B., Dolinger A., Bedoya G. (2017). Use of standardised patients to assess quality of healthcare in Nairobi, Kenya: a pilot, cross-sectional study with international comparisons. BMJ Global Health.

[19] Kwan A., Daniels B., Saria V. (2018). Variations in the quality of tuberculosis care in urban India: a cross-sectional, standardized patient study in two cities. *PLoS Med*.

[20] Christian C., Gerdtham U.G., Hompashe D., Smith A., Burger R. (2018). Measuring quality gaps in TB screening in South Africa using standardised patient analysis. *Int J Environment Res Public Health*.

[21] Rethans J., Drop R., Sturmans F., Vleuten C van der. (1991). A method for introducing standardized (simulated) patients into general practice consultations. Br J Gen Pract.

[22] Glassman P.A., Luck J., O'Gara E.M., Peabody J.W. (2000). Using standardized patients to measure quality: evidence from the literature and a prospective study. *Joint Commission J Qual Improv*.

[23] Satyanarayana S., Kwan A., Daniels B. (2016). Use of standardised patients to assess antibiotic dispensing for tuberculosis by pharmacies in urban India: a cross-sectional study. *Lancet Infect Dis*.

[24] Sylvia S., Shi Y., Xue H. (2014). Survey using incognito standardized patients shows poor quality care in China's rural clinics. *Health Policy Plan*.

[25] Schnippel K., Meyer-Rath G., Long L. (2012). Scaling up xpert MTB/RIF technology: the costs of laboratory-vs. clinic-based roll-out in South Africa. *Tropic Med Int Health*.

[26] Das J., Hammer J. (2005). Which doctor? Combining vignettes and item response to measure clinical competence. *J Develop Econ*.

[27] Das J., Hammer J. (2007). Money for nothing: the dire straits of medical practice in Delhi, India. *J Develop Econ*.

[28] Das J, Hammer J., Leonard K (2008). The quality of medical advice in low-income countries. *J Econ Perspect*.

[29] Furtwangler T., Malaviya S.A new approach to battling TB in Mumbai's crowded slums. accessed 4 June2019. https://www.pathorg/articles/a-new-approach-to-battling-tb-in-mumbais-crowded-slums/.

[30] World Health Organization. World Health Organization model list of essential *in vitro* diagnostics. http://www.who.int/medical_devices/diagnostics/WHO_EDL_2018.pdf2018.

[31] McDowell A., Pai M. (2016). Treatment as diagnosis and diagnosis as treatment: empirical management of presumptive tuberculosis in India. *Int J Tubercul Lung Dis*.

